# Enhanced Morbidity of Pectoralis Major Myocutaneous Flap Used for Salvage after Previously Failed Oncological Treatment and Unsuccessful Reconstructive Head and Neck Surgery

**DOI:** 10.1100/2012/384179

**Published:** 2012-05-03

**Authors:** Christiana Maria Ribeiro Salles Vanni, Leandro Luongo de Matos, Mário Paulo Faro Junior, Jossi Ledo Kanda, Cláudio Roberto Cernea, Lenine Garcia Brandão, Fábio Roberto Pinto

**Affiliations:** ^1^Department of Head and Neck Surgery, ABC Medical School, São Paulo, SP, Brazil; ^2^Department of General Surgery, ABC Medical School, São Paulo, SP, Brazil; ^3^Department of Head and Neck Surgery, University of São Paulo Medical School, São Paulo, SP, Brazil; ^4^São Paulo State Cancer Institute and Department of Head and Neck Surgery, University of São Paulo Medical School, São Paulo, SP, Brazil

## Abstract

*Introduction*. The reconstruction of complex cervicofacial defects arising from surgical treatment for cancer is a real challenge for head and neck surgeons, especially in salvage reconstruction surgery and/or failed previous reconstruction. The pectoralis major myocutaneous flap (PMMF) has been widely used in these specific situations due to its reliability and low rate of failure or complications. *Objectives*. Identify factors that determine complications and influence the final outcome of the reconstructions with PMMF in salvage cancer surgery or in salvage reconstruction. *Methods*. A cross-sectional study design was used to evaluate a sample including 17 surgical patients treated over a period of ten years that met the inclusion criteria. *Results*. Reconstruction was successful in 13 cases (76.5%), with two cases of partial flap loss and no case of total loss. Complications occurred in 13 cases (76.5%) and were specifically related to the flap in nine instances (52.9%). An association was identified between the development of major complications and reconstruction of the hypopharynx (*P* = 0.013) as well as in patients submitted to surgery in association with radiation therapy as a previous cancer treatment (*P* = 0.002). The former condition is also associated with major reconstruction failure (*P* = 0.018). An even lower incidence of major complications was noted in patients under the age of 53 (*P* = 0.044). *Conclusion*. Older patients, with hypopharyngeal defects and submitted to previous surgery plus radiation therapy, presented a higher risk of complications and reconstruction failure with PMMF.

## 1. Introduction

Cancer treatment of the head and neck generally results in major defects that usually cannot be repaired by primary closure or mobilization of neighboring tissues, thus requiring surgical flaps for reconstruction. The employment of flaps provides better esthetic and functional results, aiding the patients to recover its capacity of work and return to social life. There are many types of flaps available, including local, pedicled and the microsurgical flaps, which are considered the gold standard for head and neck surgery [1]. In recent decades, reconstruction with microsurgical flaps has been widely used in this type of surgery, providing better esthetic and functional results than pedicled flaps. However, their recommendation is limited by the need for professionals with extensive experience in microsurgery, specific and costly materials, and healthy clinical conditions of the patient that allow him to tolerate generally longer surgical procedures. Another factor limiting the use of microsurgical flaps is the necessity of good-quality recipient blood vessels for anastomosis [2].

With the growing number of head and neck cancer patients initially submitted to nonsurgical treatment (chemotherapy and/or radiation therapy), there has been an increase in the number of salvage surgeries where the use of microsurgical flaps is not possible, whether due to the technical issues regarding anastomosis or due to the low-performance status of the patient [3]. Frequently this situation forces the head and neck surgeon to use pedicled flaps in the reconstruction of the defects resulting from ablative oncological surgery. Among these flaps, the pectoralis major myocutaneous flap (PMMF), described by Ariyan in 1979, is the most often employed due to its reliability and versatility [4]. Complications from PMMF vary widely in the literature, where reported rates range from 13% to 63% [5], and several risk factors are described for complications and failures when this flap is employed. One of the well-recognized risky factors is the situation in which the patient has already been submitted to previous cancer treatment (surgery and/or radiation therapy). Despite the number of publications on the subject, there is little in the literature regarding salvage surgical treatment or failed reconstruction in which the PMMF is used. The lack of information on the subject served as the motivation for this study.

Thus, this research aims to analyze our casuistic of head and neck reconstructions with the PMMF in oncological salvage surgeries or failed reconstructions and identify the factors that may determine the complications and influence the final outcome of this specific type of reconstruction.

## 2. Casuistic and Methods

This was a cross-sectional study based on the analysis of patient charts carried out by the Department of Head and Neck Surgery of the ABC Medical School in the Padre Anchieta Teaching Hospital. It was approved by the Institution's Research Ethics Committee.

In the study were included 17 patients submitted to cervicofacial reconstructions using the PMMF, after salvage surgery for locoregional relapse of squamous cell carcinomas of the head and neck region and/or failed reconstruction during the period January 2002 to June 2010 at the ABC Medical School Teaching Hospital. The data were collected, the clinical oncological stage was reviewed according to 2002 TNM criteria from the *Union Internationale Contre le Cancer* (UICC), and the tumors classified in stages of I to IV.

**Figure 1:**
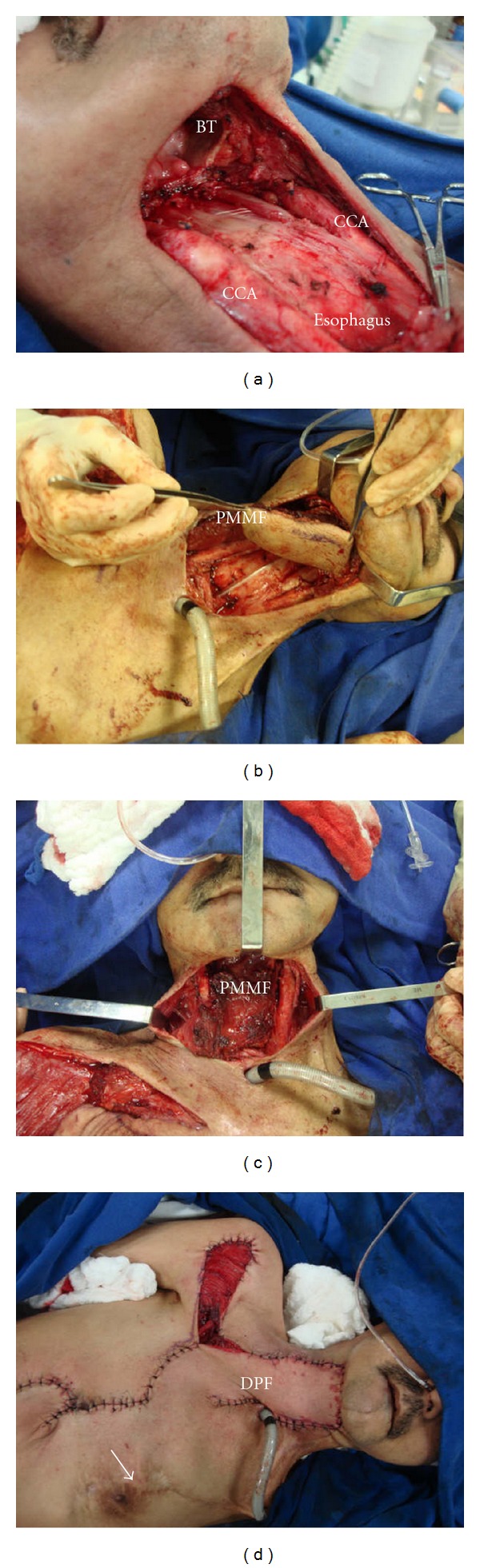
(a) Defect resulting from a circular laryngopharyngectomy for a stage IV squamous cell carcinoma in a patient previously treated with surgery and radiation therapy from an oropharynx cancer (BT: base of tongue; CCA: common carotid artery). (b) Pectoralis major myocutaneous flap (PMMF) being sutured over the pre-vertebral fascia, creating the neo-hypopharynx. (c) PMMF in place after the end of the sutures. (d) Deltopectoral flap (DF) rebuilding the neck skin and covering the PMMF (the arrow shows the chest scar from the contralateral PMMF used in the reconstruction of the defect secondary to the surgical treatment of the first primary tumor of the patient).

The surgical technique used to harvest the PMMF is described in specialized literature [1, 4, 6]. In most cases the vascular pedicle was dissected by direct visualization, by an incision extending from the upper border of the skin paddle to the midclavicular point, fully exposing the pectoralis major muscle and transposing the flap via the supraclavicular route. In one case, the PMMF was used in combination with the deltopectoral flap, as shown in Figure 1. The areas of the reconstruction were divided into three categories: skin, intraoral (oral cavity and/or oropharynx), and hypopharynx. Reconstructions of the oral cavity and oropharynx were grouped into a single category to simplify the analysis. The skin defect reconstructed by the deltopectoral flap (Figure 1) was not taken into account in the analysis of the present study.

The following postoperative variables were studied: final outcome of the reconstruction and the presence of complications and their severity. The final outcome of the reconstruction was stratified into two categories: *success*, when the objective of the reconstruction was achieved, and *failure*, when it was unsatisfactory or the patient died due to post-operative complications. For example, for defects of the oral cavity and pharynx, the objective of the reconstruction was to enable speech intelligibility and to restore swallowing, without the need for a feeding tube.

Complications were classified as *complications related to the flap*, or in other words, those directly associated with the flap or with the reconstructed area, as well as the donor site. *Other complications *are those not directly related to the repair procedure, which include clinical and surgical complications. Complications related to the flap were categorized into *major and minor complications *as suggested by Chepeha et al. [7]. Major complications were those that needed reoperation in a surgical theater under anesthesia or that resulted in failure of the reconstruction objective. Minor complications were considered those that were treated in a conservative manner, or in other words, without the need for new surgery to address repairs and that resulted in a successful reconstruction. Conservative treatment comprised bandages, small drains or debridement, and the use of medication. As also proposed by Chepeha et al. [7], when one complication led to another, only the latter was considered in an effort to represent the real outcome of the individual patient. For example, if a dehiscence resulted in an orocutaneous fistula, then the fistula was considered as a complication in the final analysis. Ischemic complications, such as partial or total loss of the flap, were analyzed separately, regardless of the other complications associated with the ischemic event. For example, if the partial loss led to dehiscence and fistula, they were then considered “partial loss” and “fistula.” In the cases in which there was some type of flap loss, we tried to identify the possible technical causes that could be associated to it. Unrelated complications were considered separately, for example, orocutaneous fistula and dehiscence of the donor site.

### 2.1. Data Analysis

In each case, the parameters “final reconstruction outcome” and “presence or absence of minor or major complications” were compared to the following variables: age stratified by the average, stage of the disease (I–IV), reconstructed site, previous cancer treatment, and need for blood transfusion. Previous cancer treatment was stratified into radiation therapy (with or without chemotherapy), surgery, or the combination of surgery and radiation therapy. All the patients submitted to reconstruction of the oral cavity, oropharynx, and hypopharynx received specialized speech therapy and swallowing rehabilitation.

The program SPSS version 17.0 (SPSS Inc; IL, USA) was used in the statistical analysis. The distributions were defined as nonparametric by the Kolmogorov-Smirnov test. The values obtained from the study of each continuous variable were organized and described using averages and standard deviation, and relative and absolute frequencies were used for categorized variables. In the comparison of frequency of the phenomenon between groups of categorized variables, Fischer's exact test and chi-squared test were used. In all analyses we considered a chance of 5% or less to commit a type I or *α* error (*P* ≤ 0.05).

## 3. Results

All 17 patients were male with an average age of 53.8 ± 9.2 years (minimum of 38 and maximum of 74 years). Most patients presented relapses diagnosed in advanced stages (mainly stage IV). The most common sites of relapse were the neck, the oral cavity, and the oropharynx. Hospital stay ranged from 2 to 32 days (average of 10.0 ± 9.6 days). The average time for harvesting and transposing the flap to the site of reconstruction was 50 minutes. The distribution of the cases according to the reconstructed area, previous cancer treatment, need for blood transfusion, and other descriptive data of the sample is shown in Table 1. Ten patients received radiotherapy (as the main treatment or after surgery) and the doses ranged from 50 to 70 Gy. We did not take into account the radiotherapy dose as another variable due to the small number of patients of our casuistic.

*Success* of reconstruction was achieved in 13 cases (76.5%), and *failure *was the outcome in the other four cases. All of these four patients presented cervical dehiscence: three complicated by pharyngocutaneous fistula and orocutaneous fistula. Two of these patients could not be rehabilitated due to these complications, and the other two died: one due to the exposure and rupture of large cervical vessels during the 31st post-operative day and the other due to bronchopneumonia after the 12th post-operative day. We identified two cases of partial necrosis of the flap (11.8%). The first case developed approximately 50% of skin paddle necrosis due to a hematoma of the donor site with compression of the vascular pedicle. The second case presented less than 25% of skin paddle necrosis, due to the skin island of the flap was located beyond the edge of the seventh rib, making this portion of the flap random. In the first case, failure was the final outcome of the reconstruction due to the death of the patients from surgical complications, and in the second case success with conservative treatment of the dehiscence resulting from ischemia. There was no case of total flap loss.

Some type of complication (Table 2) occurred in 13 cases (76.5%), and complications related to the flap were observed in nine cases (52.9%). Major complications occurred in five patients (29.4%) and minor complications in four (23.5%). Other complications were observed separately or in association in seven cases (41.2%). In a comparison of the variables, a statistically significant association was identified between the development of major complications (Table 3) and the reconstruction of the hypopharynx (*P* = 0.013—chi-squared test; all three cases of reconstruction of the hypopharynx presented major complications) and also in patients submitted to surgery in association with radiation therapy as a previous cancer treatment (*P* = 0.002—chi-squared test; all four cases of patients previously submitted to surgery in association with adjuvant radiation therapy presented major complications). A lower incidence of major complications was noted in patients under the age of 53 (*P* = 0.044—Fischer's exact test). Other differences were not identified when compared to the stage of neoplasia and need for blood transfusion.

With regard to the success of reconstruction (Table 4), the statistically significant difference observed was a higher chance of failure in the reconstruction when the patient had been previously submitted to surgery followed by radiation therapy (*P* = 0.018—chi-squared test; three out of four cases previously submitted to surgery and radiation therapy—75%—presented failure as the final outcome of reconstruction). For the other variables, no significant association was observed.

## 4. Discussion

Despite recent advances in microsurgical techniques, pedicled flaps are still an acceptable option for the reconstruction of complex defects of the head and neck, especially intraoral defects and those of the hypopharynx and skin [8]. Various types of treatment for the relapse of head and neck cancer have been studied and discussed, mainly emphasizing radical resection and immediate reconstruction. Among the reparative techniques available for salvage procedures, PMMF is the most reliable and versatile and may be used as the first choice for patients that have been consumed by cancer or sufferers of severe comorbidity. In these situations, a flap that, in addition to being reliable, requires shorter surgical time is recommended [2].

At our department (ABC Medical School), the PMMF was the main alternative for reconstruction of large cervicofacial defects up until 2004. Before this date the technical conditions were not suitable for the use of microsurgical flaps. After the introduction of free flaps, we reserved the PMMF for those situations in which the microsurgical technique is not recommended or not advisable, that is, in patients clinically compromised or when the local conditions of the receptor vessels of the neck are not appropriate for vascular anastomosis. Upon analyzing our cases, we observed that the PMMF was used for the reconstruction of secondary defects to the resection of tumors located in different sites, which attests to the versatility of the PMMF, widely cited in the literature. The election of the PMMF as the standard flap of our department for salvage reconstructions is due to the characteristics of the flat itself, previously described, like the ease to harvest and its reliability, attested to by the reduced number of losses, low rate of complications, and high rate of success. The literature shows that the PMMF produces good results [9, 10], although with varied rates of complications (13% and 63%) [5, 6, 11–20], generally in patients already submitted to previous oncological treatment (surgery and/or radiation therapy), which include fistula, dehiscence, infection, and hematoma [20, 21]. 

Ischemic complications and consequent necrosis are described in up to 32% of the cases [20, 22], which in general present necrosis and partial loss of the flap and do not require another surgical repair procedure; total loss of the PMMF is infrequent, described in isolated cases, and oftentimes related to technical issues [1]. In a study recently published [1], our group observed a reconstruction success rate of 93.1%, with 12% presenting partial loss (all of these partial loss cases were successfully reconstructed) and no case of total flap loss was recorded.

Various factors are described as related to complications when PMMF is used, especially in salvage surgeries. McLean et al. [11] described more complications in previously irradiated patients, El-Marakby [13] established an association between the higher number of comorbidities and reconstruction of the oral cavity, and Zbar et al., [17] in addition to the factors already mentioned, added surgeries carried out to cover bone due to osteoradionecrosis. Other authors pointed out smoking and diabetes as being associated with a higher incidence of complications [13, 14, 16].

Chiummariello et al. [3] studied the complications of salvage reconstructions for the oral cavity, oropharynx, skin and hypopharynx due to squamous cell carcinoma in 12 patients over a period of 17 months. They identified that there was no case of total flap loss and that successful reconstruction was achieved in 100% of the cases. Complications were also found in 33% of the cases, including partial loss, infection, and dehiscence in 8% and orocutaneous fistula in 16% of the cases. The authors found multiple significant variables related to the development of complications: over 65 years of age, male sex, smoking and alcoholism, location of the tumor in the hypopharynx, reconstruction of the hypopharynx, advanced stage of the primary tumor, comorbidities (diabetes, hypertension, and arterial sclerosis), low albumin and hemoglobin, and previous radiation therapy on the site to be reconstructed. These findings corroborate the results found in the present study, where the patients with the highest risk of complications were those over 53 years of age and those needing reconstruction of the hypopharynx.

Xiao et al. [23] retrospectively studied 31 cases of reconstructions of the hypopharynx and cervical esophagus due to relapse of carcinoma of the hypopharynx and larynx over a period of 11 years, of which there were effectively five cases of PMMF in salvage reconstructions, three for the hypopharynx, one for the base of the tongue, and one for the cervical skin. The average age of patients was 61.4 years (ranging from 40 to 76 years), 93.5% of the patients were male, and complications were observed in 22.6% of the patients, where pharyngocutaneous fistula and dysphagia were the most prevalent. The authors also report a death due to the rupture of the carotid as the result of cervical dehiscence and pharyngocutaneous fistula. This fact was also observed in our study, since there was one death due to post-operative complications; however, cervical dehiscence with the exposure of vessels requiring reoperation was not observed.

Kruse et al. [5] performed 20 cases of PMMF over 11 years, of which seven were salvage surgeries (five for the mandible and two for the floor of the mouth), all with previous radiation therapy. For these specific cases the rate of complications was 42.8%, with 30% partial loss of the flap, and one case of total necrosis of the flap and failure of the reconstruction. In the casuistic herein presented, there was no case of total flap loss and four cases of failed reconstruction (23.5%).

McLean et al. [11]. in a 16-year series, studied the results of 136 reconstructions using PMMF, of which 46 cases were salvage reconstructions. The authors mentioned that PMMF was the definitive technique for reconstruction in all patients. The overall rate of complications of this study was 13%, with four cases of fistula (three in cases previously irradiated) due to partial flap loss or dehiscence of the wound and one case of total flap loss treated with contralateral PMMF. In the present study there was no need for harvesting the contralateral PMMF to cover occasional dehiscence or partial loss of PMMF. Zou et al. [24] studied 24 cases of PMMF employed in salvage surgeries for relapse of squamous cell carcinoma of the oral cavity and oropharynx (8 post-operative cases associated with radiation therapy, 4 isolated afterradiation therapy cases, and 12 cases after surgery alone). Successful reconstruction was achieved in 70.8% of the cases, and the rate of complications was 62.5%, with three cases of marginal necrosis of the flap, three dehiscences of the operative wound, five cases of necrosis of up to 40% of the flap, and two cases of necrosis beyond 40% of the flap. The following variables were related to a higher rate of complications: male sex, over 50 years of age, radiation therapy of over 30 Gy, duration of surgery of over 7.3 hours, presence of comorbidities (hypertension and diabetes), flaps larger than 42 cm^2^, alcoholism, smoking, advanced stages (T3 or T4) and subsite of reconstruction being the tongue, floor of the mouth, or the oropharynx. Among these factors, only the latter item emerged as an independent variable that determines risk of complications by multivariate logistic regression.

Finally, it is important to stress that, in the present study, unlike other studies in the literature, both salvage reconstructions and oncological salvage surgeries were included. We therefore describe our casuistic as being composed of previously treated patients, which makes our study novel. Despite the limited number of cases studied, over a relatively short period (10 years), we obtained a sample sufficient to conclude that older patients, with defects of the hypopharynx and submitted to previous radiation therapy and surgery, present a greater risk of complications and failure in reconstruction with PMMF. We therefore recommend that, in salvage surgeries, whether oncological salvage or salvage reconstruction, in which the conditions described above are observed, strategies must be adopted in the harvesting of the PMMF with a view to treatment of occasional complications or failures. Among these strategies is the preservation of the deltopectoral flap during elevation of the PMMF, in order to reserve this other flap in the event of failure of the PMMF. Another important policy is to avoid any situation that could place the viability of the PMMF at risk such as including random areas in the skin island or placing it outside the area of its perforating vessels.

## Figures and Tables

**Table 1:** Descriptive data.

Characteristic	Result
Males**	17 (100%)
Age (years)	53.8 ± 9.2 (38–74)
Duration of hospital stay (days)*	10.0 ± 9.6 (2–32)
Stage**	
I	0 (0%)
II	2 (11.8%)
III	1 (5.9%)
IV	14 (82.4%)
Area reconstructed**	
Skin	7 (41.2%)
Intraoral	7 (41.2%)
Hypopharynx	3 (17.6%)
Previous oncological treatment	
Radiation therapy (with or without chemotherapy)	6 (35.3%)
Surgery alone	7 (41.2%)
Surgery combined with adjuvant radiation therapy	4 (23.5%)

* Average ± standard deviation (minimum-maximum).

** Absolute numbers (percentage).

**Table 2:** Observed complications.

Characteristic	Result
Major complications	5/17 (29.4%)
Cervical dehiscence	2
Pharyngocutaneous fistula	3

Partial loss	1
Death	2

Minor complications	4/17 (23.5%)
Cervical dehiscence	2
Pharyngocutaneous fistula	1
Orocutaneous fistula	1

Partial loss	1

Other complications	7 (41.2%)
Anemia	4
Pneumonia	1
Severe renal insufficiency	1
Cervical abscess	1
Electrolyte disturbances + hypertension crisis	1

**Table 3:** Analysis of the variables determining major complications resulting from reconstruction.

Characteristic	Major complication*		Significance
	Yes	No	
Age			
<53 years	0 (0.0)	7 (58.3)	*P* = 0.044^□∗∗^
≥53 years	5 (100.0)	5 (41.7)	
Stage			
II	0 (0.0)	2 (16.7%)	*P* = 0.468^■^
III	0 (0.0)	1 (8.3%)	
IV	5 (100.0)	9 (75.0%)	
Area reconstructed			
Skin	1 (20.0)	6 (50.0)	*P* = 0.013^■∗∗^
Intraoral	1 (20.0)	6 (50.0)	
Hypopharynx	3 (60.0)	0 (0.0)	
Previous treatment			
Radiation therapy	0 (0.0)	6 (50.0)	*P* = 0.002^■∗∗^
Surgery	1 (20.0)	6 (50.0)	
Surgery + radiation therapy	4 (80.0)	0 (0.0)	
Need for blood transfusion			
Yes	2 (40.0)	2 (16.7)	*P* = 0.538^□^
No	3 (60.0)	10 (83.3)	

Legend: □ Fischer's exact test. ■ Chi-squared test. *Absolute number (%). **P ≤ 0.05 indicates statistical significance.

**Table 4:** Analysis of the variables determining failure in reconstruction.

Characteristic	Success in reconstruction*		Significance
	Yes	No	
Age			
<53 years	7 (53.8)	0 (0.0)	*P* = 0.103^□^
≥53 years	6 (46.2)	4 (100.0)	
Stage			
II	2 (15.4)	0 (0.0)	*P* = 0.571^■^
III	1 (7.7)	0 (0.0)	
IV	10 (76.9)	4 (100.0)	
Area reconstructed			
Skin	6 (46.2)	1 (25.0)	*P* = 0.152^■^
Intraoral	6 (46.2)	1 (25.0)	
Hypopharynx	1 (7.6)	2 (50.0)	
Previous treatment			
Radiation therapy	6 (46.2)	0 (0.0)	*P* = 0.018^■∗∗^
Surgery	6 (46.2)	1 (25.0)	
Surgery + radiation therapy	1 (7.6)	3 (75.0)	
Need for blood transfusion			
Yes	2 (50.0)	2 (15.4)	*P* = 0.219^□^
No	2 (50.0)	11 (84.6)	

Legend: □ Fischer's exact test. ■ chi-squared test. *Absolute number (%). **P ≤ 0.05 indicates statistical significance.
